# Soman induces endoplasmic reticulum stress and apoptosis of cerebral organoids via the GRP78‐ATF6‐CHOP signaling pathway

**DOI:** 10.1002/2211-5463.70027

**Published:** 2025-03-28

**Authors:** Yue Wei, Zhanbiao Liu, Jingjing Shi, Qian Jin, Wenqian Chen, Xuejun Chen, Liqin Li, Hui Chen

**Affiliations:** ^1^ College of Pharmacy Guilin Medical University China; ^2^ State Key Laboratory of NBC Protection for Civilian Beijing China; ^3^ Key Laboratory of Pharmacology for Prevention and Treatment of High Incidence Diseases in Guangxi Higher Education Institutions Guilin Medical University China

**Keywords:** ATF6, cerebral organoids, ER stress, organophosphates, soman

## Abstract

Soman is an organophosphorus compound that induces neurotoxicity. In addition to its direct toxic effects resulting from acetylcholine accumulation, neurotoxicity may also be exacerbated by inducing endoplasmic reticulum (ER) stress. In light of the current scarcity of appropriate *in vitro* assessment models, in the present study, we used cerebral organoids derived from human pluripotent stem cells, a new tool for investigating the mechanisms of neurotoxicity, to investigate soman‐induced ER stress. The results demonstrated that soman significantly suppressed acetylcholinesterase activity and activated the GRP78‐ATF6‐CHOP (i.e. glucose‐regulated protein 78‐activating transcription factor 6‐C/EBP homologous protein) ER stress cascade, driving apoptosis in cerebral organoids. Pharmacological inhibition of ER stress by pre‐treating cerebral organoids with the ER stress inhibitor 4‐phenylbutyric acid prior to soman exposure attenuated apoptotic signaling and downregulated GRP78, ATF6 and CHOP expression. Parallel *in vivo* validation utilized a rat model with subcutaneous soman exposure, focusing on hippocampal and striatal ER stress markers. Consistent with the *in vitro* findings, soman‐exposed rats exhibited marked ER stress activation in brain regions critical for neurotoxicity. This study establishes ER stress as a key contributor to soman‐induced neurotoxicity and highlights cerebral organoids as a physiologically relevant model for organophosphorus compound research. We propose ER stress modulation as a potential therapeutic strategy to mitigate neurotoxic outcomes.

## Graphical abstract

Cerebral organoids were employed as a novel model to explore the neurotoxicity of soman. Soman inhibited acetylcholinesterase activity, increased cell apoptosis and upregulated endoplasmic reticulum (ER) stress markers glucose‐regulated protein 78 (GRP78), activating transcription factor 6 (ATF6) and C/EBP homologous protein (CHOP). The ER stress inhibitor 4‐phenylbutyric acid prevented apoptosis and reduced ER stress‐related protein induced by soman. These findings suggest ER stress modulation as a promising neuroprotective strategy against organophosphorus poisoning.

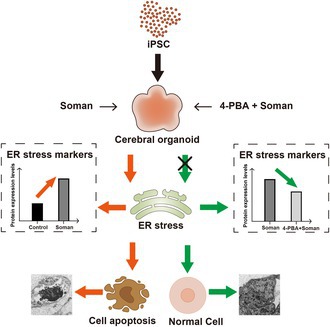

**Abbreviations**
4‐PBA: 4‐phenylbutyric acid
ACh: acetylcholine
AChE: acetylcholinesterase
ATF6: activating transcription factor 6
ANOVA: analysis of variance
CHOP: C/EBP homologous protein
ER: endoplasmic reticulum
GRP78: glucose‐regulated protein 78
iPSC: induced pluripotent stem cell
IRE1: inositol‐requiring enzyme 1
OP: organophosphorus
PERK: protein kinase RNA‐like endoplasmic reticulum kinase
qPCR: quantitative real‐time PCR
TEM: transmission electron microscope
TUNEL: terminal deoxynucleotidyl transferase–mediated dUTP nick end labeling
UPR: unfolded protein response

Organophosphorus (OP) compounds are a class of highly hazardous nerve agents known for their neurotoxicity, carcinogenicity and secretory toxicity [1, 2]. OPs such as soman were used as chemical warfare agents during World War II and are still employed in various industries, including as flame retardants, plasticizers, engine oil additives, medicines and pesticides [3]. OPs typically disrupt the transmission of acetylcholine (ACh) by inhibiting acetylcholinesterase (AChE) [4]. This inhibition leads to an accumulation of ACh, causing muscarinic and nicotinic neurotoxicity and other clinical symptoms, ultimately resulting in severe dysfunction of the central and peripheral cholinergic nervous systems [5]. Despite extensive evidence of neuropathological and behavioral impairments following acute and chronic OP exposure in animals and humans, the exact mechanisms remain poorly understood [6, 7]. Endoplasmic reticulum (ER) stress is a protective cellular mechanism that is activated by the accumulation of misfolded or unfolded proteins. This condition impairs the ability of the ER to fold proteins correctly, which can result from various internal or external stimuli [8, 9]. Consequently, this impairment can lead to cell death and the activation of pro‐inflammatory proteins and apoptotic pathways [10].

Several studies have linked ER stress to OP‐induced cytotoxicity. Gur and Kandemir [11] reported upregulation of ER stress markers, such as activating transcription factor 6 (ATF6), protein kinase RNA‐like endoplasmic reticulum kinase (PERK), inositol‐requiring enzyme 1 (IRE1), glucose‐regulated protein 78 (GRP78) and proapoptotic factor C/EBP homologous protein (CHOP), in the liver and kidney of rats administered the pesticide malathion. OP esters have been found to induce reactive oxygen species overproduction, DNA damage and ER stress in human hepatocellular carcinoma cells [12]. In mouse primary cortical neuronal cultures, chlorpyrifos enhanced the expression of ER stress‐related genes and proteins [13]. However, there are few reports on ER stress induced by nerve agents, comprising a group of the most toxic OP compounds. It remains unclear whether soman induces ER stress and whether ER stress contributes to soman‐induced cell apoptosis.

Most existing data derive from 2D cell cultures or animal studies, which have limited extrapolative value to humans due to the complexity of human brain tissues and species differences [14, 15, 16]. Cerebral organoids, derived from human induced pluripotent stem cells (iPSCs) or embryonic stem cells, showing more accurately reflect human brain development, tissue composition, and physiological processes compared to conventional 2D cultures [17]. This technology has significant translational potential for mimicking human neurodevelopment and disorders and provides an excellent tool for studying the toxicity and mechanisms of toxicants [18, 19]. Recent research has demonstrated the feasibility of using cerebral organoids to investigate the neurotoxicity of chemicals and nanomaterials, including rotenone [20], tranylcypromine [21], acrylamide [22], silver nanoparticles [23] and zinc oxide nanoparticles [24].

In the present study, we constructed iPSC‐derived cerebral organoids to investigate the molecular mechanisms underlying soman‐induced ER stress. We assessed ER stress markers in soman‐damaged cerebral organoids at both the mRNA and protein levels. Subsequently, we utilized rats to confirm the activation of ER stress following soman exposure. Our results demonstrated that soman induces ER stress by activating the ATF6/GRP78/CHOP pathway.

## Materials and methods

### Animal grouping, exposure and sample preparation

Adult male Sprague–Dawley rats (weighing 190–210 g, aged 8 weeks) were purchased from Beijing HFK Bioscience Co., Ltd (Beijing, China) and were housed in ventilated cages consisting of four rats together under a 12 : 12 h light/dark photocycle, at 20–25 °C and 40–70% relative humidity, with water and standard rat chow (Beijing HFK Bioscience Co., Ltd) freely available. The control group received a subcutaneous injection of normal saline, whereas the soman‐treated group received a single subcutaneous injection of LD_50_, which is 98 μg·kg^−1^ soman for 0.5, 6 and 24 h. All rats were euthanized in a carbon dioxide anesthesia box and whether the heartbeat had stopped completely was checked. Hippocampus and striatum were collected from each rat at the end of each time point. Total RNA was extracted from brain tissues using the RNeasy Mini Kit (Qiagen, Valencia, CA, USA) (*n* = 6). Furthermore, the hippocampus and striatum were homogenized using RIPA lysis buffer (Solarbio, Beijing, China). Following dissociation on ice and centrifugation at 12 000 ***g*** at 4 °C for 15 min, the protein supernatant was collected and set aside (*n* = 4) The animal study protocol was approved by the Guiding Principles for the Animal Ethics Committee of the State Key Laboratory of NBC Protection for Civilian (approval no. LAE‐2023‐06‐004).

### Human iPSC culture and expansion

Human iPSCs, reprogrammed from the urine cells of a 28‐year‐old woman, were provided by CellApy Biotechnology (Beijing, China). These cells were cultured in flasks coated with a bedding solution (CellApy Biotechnology) using mTeSR Plus medium (STEMCELL Technologies, Vancouver, BC, Canada). When confluence reached 80%, iPSCs were gently dissociated and subcultured using gentle cell dissociation reagent (STEMCELL Technologies).

### Generating cerebral organoids from iPSCs

Cerebral organoids were generated using the STEMdiff™ Cerebral Organoid Kit (STEMCELL Technologies) in accordance with the manufacturer's instructions. Briefly, a single‐cell suspension of iPSCs in mTeSR Plus medium with 10 μmol·L^−1^ Y‐27632 (Selleck, Houston, TX, USA) was added to a 96‐well Clear Round Bottom Ultra‐Low Attachment Microplate (Thermo Fisher, Waltham, MA, USA) with 2000 cells per well. After low‐speed centrifugation, the plate was incubated at 37 °C in a CO_2_ incubator (Thermo Fisher) for 24 h. On day 5, embryoids were transferred to induction medium. On day 7, embryoids were embedded in Matrigel (Corning, New York, NY, USA) droplets and transferred to an ultra‐low adhesion six‐well plate (Corning) containing expansion medium. On day 10, the cultures were replaced with maturation medium and transferred to an orbital shaker or rotating reaction flask for long‐term culture. Mature cerebral organoids, approximately 40 days old, were used for subsequent experiments.

### Soman and 4‐phenylbutyric acid (4‐PBA) exposure

Soman (CAS no. 96‐64‐0, > 95%) was provided by the Laboratory of Analytical Chemistry, Research Institute of Chemical Defense (Beijing, China). Mature cerebral organoids were treated with 200 nmol·L^−1^ soman for various durations (0.5, 6 and 24 h) by mixing soman into the culture medium, followed by washing three times with phosphate‐buffered saline (*n* = 3). For AChE activity detection, cerebral organoids were exposed to soman at concentrations of 6.25, 25, 50, 100 and 200 nmol·L^−1^, with three organoids per concentration. For immunofluorescence and the terminal deoxynucleotidyl transferase–mediated dUTP nick end labeling (TUNEL) assay, cerebral organoids were treated with 200 nmol·L^−1^ soman for 24 h, with three organoids per group. For quantitative real‐time PCR (qPCR) and western blot analysis, cerebral organoids were divided into four groups with three organoids per group. The control group was exposed to 0.1% solvent, whereas the other groups were exposed to 200 nmol·L^−1^ soman for 0.5, 6 and 24 h, respectively. In addition, 10 mmol·L^−1^ 4‐PBA (MedChemExpress, Monmouth Junction, NJ, USA) was used to treated mature cerebral organoids for 1 h before soman exposure.

### AChE activity

Cerebral organoids, 40 days old, were transferred to an ultra‐low adhesion 96‐well plate (Corning) with one organoid per well. The organoids were exposed to a gradient concentration of soman (0, 6.25, 25, 50, 100 and 200 nmol·L^−1^) with three organoids per concentration. After 10 min of incubation at 37 °C, the organoids were transferred to Eppendorf tubes, washed with phosphate buffer, homogenized with an electric grinder (Tiangen, Beijing, China) at 2500 ***g*** for 30 s on ice, and centrifuged at 2500 ***g*** for 10 min. AChE activity in the supernatant was determined using a modified Ellman's method in a 96‐well plate. The detection system contained 0.3 mmol·L^−1^ 5,5′‐dithiobis‐(2‐nitrobenzoic acid) (Sigma‐Aldrich, St Louis, MO, USA), 0.45 mmol·L^−1^ acetylthiocholine (Sigma‐Aldrich) and 150 μL of sample. Absorbance at 412 nm was measured continuously at 1‐min intervals for 30 min at 37 °C.

### Immunofluorescence

For iPSCs, treated culture dishes (Corning) were used to culture the iPSCs. Cells were fixed with 4% formaldehyde (Solarbio), permeated with Triton X‐100 (Beyotime, Shanghai, China) and blocked with bovine serum albumin (Beyotime). Primary antibodies SOX2 and OCT4 (Abcam, Cambridge, UK) were applied and incubated overnight at 4 °C. Dylight 488 Goat Anti‐Rabbit IgG (Abbkine, Wuhan, China) and Alexa Fluor 594 Goat Anti‐Mouse IgG (Bioss, Beijing, China) were used as secondary antibodies, incubated for 1 h at room temperature. Nuclei were stained with DAPI (i.e. 4',6‐diamidino‐2‐phenylindole) (Beyotime) and observed under a confocal laser microscope (Leica, Wetzlar, Germany) at 10× magnification.

For cerebral organoids, the samples were fixed with 4% formaldehyde and incubated overnight at 4 °C in 15% and 30% sucrose (ACMEC, Shanghai, China), respectively. Organoids were sliced into 20‐μm sections using a cryostat (Leica). Sections were permeated and blocked, then stained with primary antibodies GALC, GFAP, MAP2, β‐III tubulin, FOXG1 and reelin (Abcam), as well as TBR1, PAX6, CTIP and SATB2 (Invitrogen, Waltham, MA, USA). Secondary antibodies, Dylight 488 Goat Anti‐Rabbit IgG and Alexa Fluor 594 Goat Anti‐Mouse IgG, were applied. Nuclei were stained with DAPI (Beyotime) and detected using a confocal laser microscope at 10× magnification. The catalog numbers and dilution ratio for antibodies are shown in Table S1.

### TUNEL assay

Cerebral organoids were exposed to soman (200 nmol·L^−1^) or 0.1% dimethylsulfoxide for 24 h, or 4‐PBA (10 mmol·L^−1^) for 1 h and then soman (200 nmol·L^−1^) for 24 h. Frozen slices were prepared as previously described. The TUNEL assay kit (Beyotime) was used to assess cell apoptosis in the cerebral organoids. After permeabilization with Triton X‐100, TUNEL detection solution was added to the slices and incubated at 37 °C for 1 h in the dark. Apoptosis was then examined using a fluorescence microscope at 10× magnification.

### Transmission electron microscopy (TEM)

Cerebral organoids were fixed by 2% glutaraldehyde for 2 h. After washing with 0.1 m sodium cacodylate buffer, samples were fixed by potassium ferrocyanide‐reduced 1% osmium tetroxide for 2 h. A gradient concentration of ethanol is used for samples dehydration. After acetone embedded, samples were cut and stained using uranyl acetate and lead citrate, and then imaged using a Talos F200X G2 TEM at 4800× magnification (Thermo, Waltham, MA, USA).

### Western blot

RIPA lysis buffer was added to the tissues or organoids, which were then homogenized using a mechanical homogenizer, lysed on ice, and centrifuged at 12 000 ***g*** at 4 °C for 15 min. Protein concentration was determined using BCA Protein Assay Kits (Thermo Fisher). Twenty micrograms of protein from each sample were separated by SDS/PAGE and transferred to poly(vinylidene difluoride) membranes (Merck, Darmstadt, Germany). Membranes were incubated overnight at 4 °C with various primary antibodies, followed by incubation with corresponding secondary antibodies for 1–2 h. Protein bands were imaged using the FluorChem E System (Protein Simple, Ottawa, ON, Canada).

### qPCR

Total RNA from brain tissues or organoids was isolated using the RNeasy Mini Kit (Qiagen). cDNA was synthesized through reverse transcription (Qiagen) and analyzed by qPCR using SYBR Green. Reactions were performed on an ABI Prism 7900HT sequence detection system (Applied Biosystems, Foster City, CA, USA). Primer sequences are listed in Table S2.

### Statistical analysis

Statistical analysis was conducted using prism, version 9.0 (GraphPad Software Inc., San Diego, CA, USA). Data are presented as the mean ± SEM. All data were simultaneously put through the Shapiro–Wilk test. Significant differences between two groups were analyzed using an unpaired *t*‐test, whereas one‐way analysis of variance (ANOVA) followed by the Tukey's test was employed to investigate differences among more than two groups. *P* < 0.05 was considered statistically significant.

## Results

### Construction and identification of cerebral organoids

**Fig. 1:**
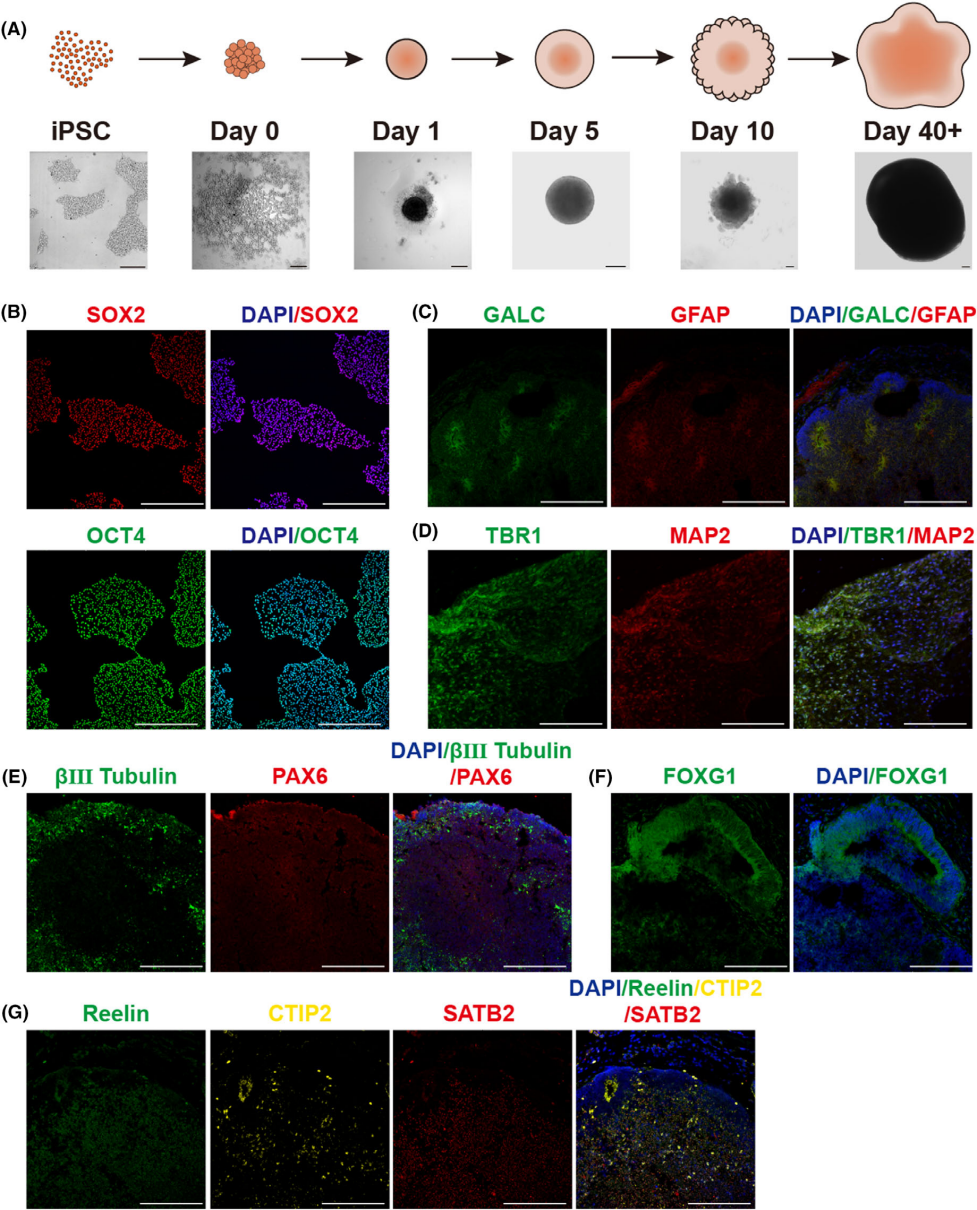
Characterization of iPSCs and cerebral organoids. (A) Showing the process of generating cerebral organoids from iPSCs. Scale bar = 200 μm. (B) Characterization of iPSCs by immunofluorescence staining of pluripotent stem cell markers SOX2 and OCT4. Scale bar = 200 μm. (C–G) Characterization of iPSCs‐derived cerebral organoids. The mature organoids showed the differentiated cells and organization structures. (C) GALC positive oligodendrocyte and GFAP positive astrocyte. (D) TBR1 positive immature neurons and MAP2 positive mature neurons. (E) β‐III tubulin positive neurons and PAX6 positive neural epithelial progenitor cells. (F) FOXG1 positive marked forebrain. (G) Reelin positive neurons indicated the surficial cortex. CTIP and SATB2 displayed early born (CTIP2) and late born (SATB2) neurons. *n* = 3. Scale bar = 200 μm.

Human iPSCs were grown in clusters, displaying a spindle shape and clear cell boundaries. Immunofluorescence staining confirmed the presence of pluripotent markers SOX2 and OCT4, indicating stem cell characteristics (Fig. 1B). The diameter of cerebral organoids derived from iPSCs exceeded 3 mm, accompanied by the generation of neuroepithelial stem cell box neurons. Surrounding epithelial structures in embryoids were observable by day 10 (Fig. 1A). Immunofluorescence identified specific markers in mature cerebral organoids, revealing positive staining for the oligodendrocyte biomarker GALC and the astrocyte biomarker GFAP (Fig. 1C).

TBR1 and βIII‐tubulin indicated the presence of immature neurons, whereas significant MAP2 expression denoted a large number of mature neurons (Fig. 1D,E). PAX6 staining indicated neural epithelial progenitor cells. Different cortical positions within cerebral organoids were identified by staining with reelin, CTIP and SATB2 (Fig. 1G). Reelin marked the superficial cortex, whereas CTIP2 and SATB2 marked early and late‐born neurons, respectively. FOXG1 staining indicated forebrain formation (Fig. 1F).

### Effects of soman on AChE activity and cell apoptosis in cerebral organoids

**Fig. 2:**
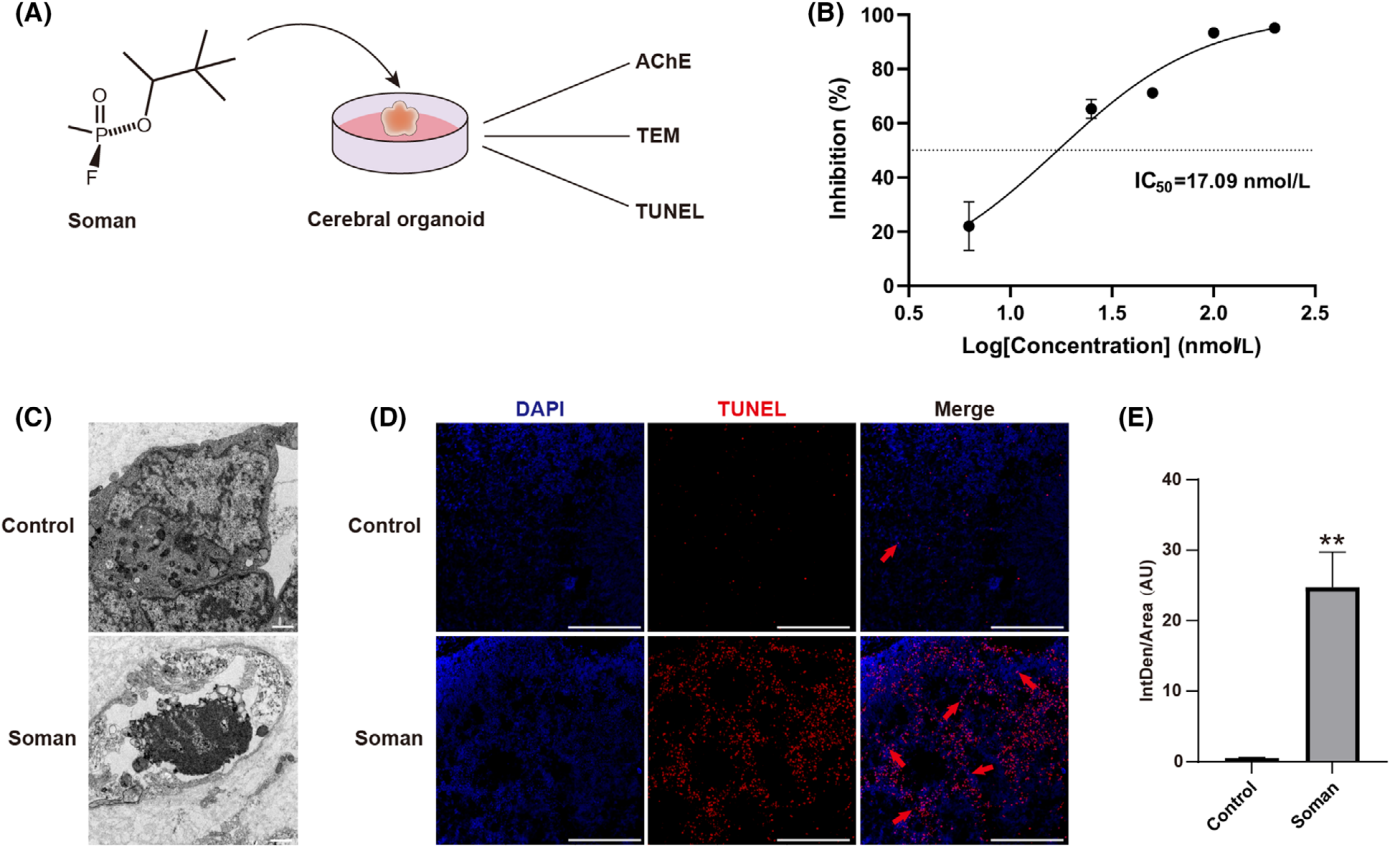
Effect of soman on AChE activity and cell apoptosis in cerebral organoids. (A) Schematic diagram of soman exposure. (B) Effects of soman on AChE activity in cerebral organoids. (C) TEM revealed cell apoptosis after soman exposure. Scale bar = 1 μm. (D) TUNEL staining shows that apoptosis cells (red) increased after soman exposure compared to the control group. Scale bar = 200 μm. (E) Quantitative analysis of TUNEL staining. Data are represented as the mean ± SEM and *n* = 3 for each group. Statistical analysis utilized unpaired *t*‐test. **P < 0.01 *vs*. control group.

Reduced AChE activity was observed in cerebral organoids exposed to varying doses of soman, with an IC_50_ of 17.09 nmol·L^−1^ and IC_95_ of 200.42 nmol·L^−1^ (Fig. 2A,B). After soman exposure, cell apoptosis was observed by TEM (Fig. 2A,C). Furthermore, TUNEL staining revealed a significant increase in apoptotic cells after soman exposure (Fig. 2A,D), with mean fluorescence intensity increasing from 0.43 to 19.33 AU compared to the control group (Fig. 2E). These results indicate that soman induces cell apoptosis, demonstrating its neurotoxicity in cerebral organoids.

### ER stress in cerebral organoids induced by soman

**Fig. 3:**
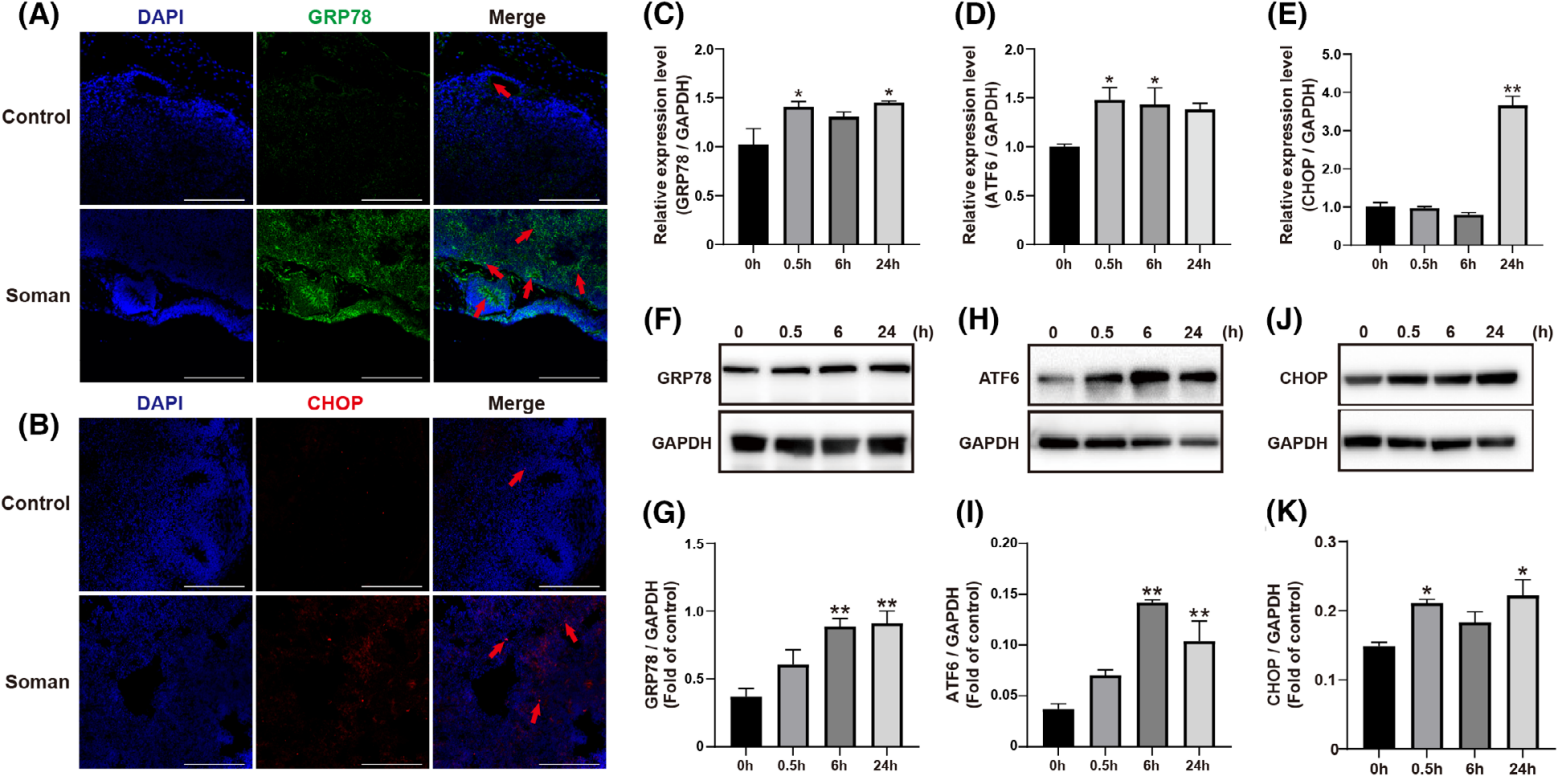
Soman activates ER stress in cerebral organoids. (A, B) Immunofluorescence for GRP78 (green) and CHOP (red) in cerebral organoids after soman exposure for 24 h. Scale bar = 200 μm. (C–E) Changes in mRNA expression of ER stress related genes GRP78, ATF6 and CHOP induced by soman in cerebral organoids. (F–J) Images of western blots for GRP78, ATF6 and CHOP. (G–K) Quantitative analysis of data from western blots. Data are represented as the mean ± SEM, *n* = 3 for each group. Statistical analysis utilized one‐way ANOVA followed by the Tukey's test. *P < 0.05, **P < 0.01 *vs*. control group.

Immunofluorescence was used to detect specific ER stress markers in cerebral organoids. Compared to the control group, the expression of GRP78 (Fig. 3A) and CHOP (Fig. 3B) significantly increased after 6 h of exposure to 200 nmol·L^−1^ soman.

ER stress‐related mRNAs in cerebral organoids were also measured. Organoids were treated with 200 nmol·L^−1^ soman for 0.5, 6 and 24 h, followed by total RNA extraction and qPCR analysis. GRP78 mRNA levels significantly increased at 0.5 and 24 h (Fig. 3C), ATF6 mRNA levels increased significantly at 0.5 and 6 h (Fig. 3D), and CHOP mRNA levels were significantly upregulated at 24 h (Fig. 3E). These results indicate ER stress activation induced by soman in cerebral organoids.

Western blot analysis further confirmed ER stress induction. Organoids treated with 200 nmol·L^−1^ soman for 0.5, 6 and 24 h showed significantly increased levels of GRP78, ATF6, and CHOP proteins (Fig. 3F–K). GRP78 and ATF6 proteins significantly increased at 6 and 24 h, whereas CHOP protein levels increased significantly at 0.5 and 6 h.

### 4‐PBA reduced cell apoptosis induced by soman in cerebral organoids

**Fig. 4:**
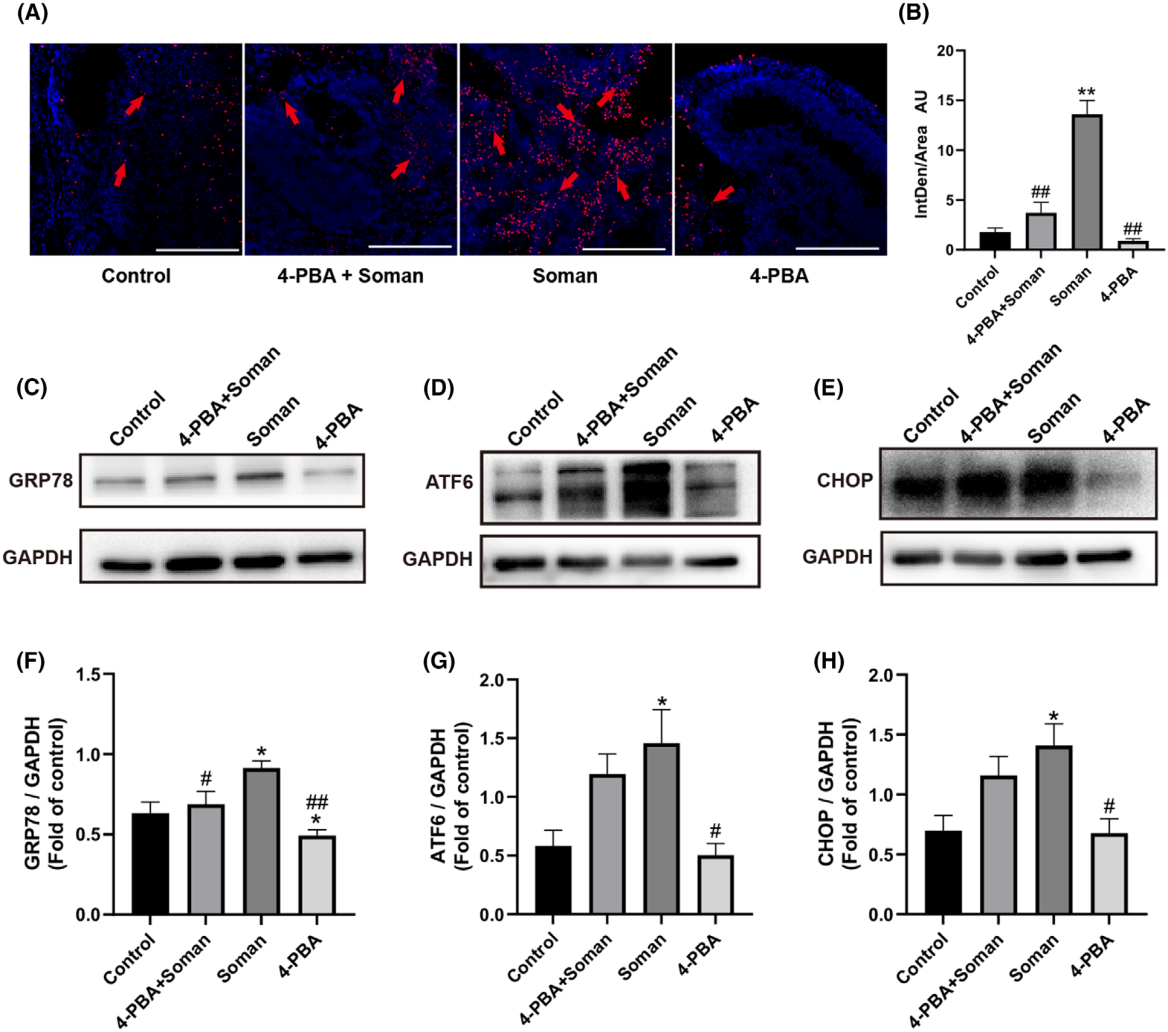
4‐PBA protected from cell apoptosis in cerebral organoids by inactivating ER stress. (A) TUNEL stain for soman exposure and 4‐PBA intervention before soman exposure. Scale bar = 200 μm. (B) Quantitative analysis of TUNEL staining. (C–E) Images of western blots for GRP78, ATF6 and CHOP. (F–H) Quantitative analysis of data from western blots. Data are represented as the mean ± SEM, *n* = 3 for each group. Statistical analysis utilized one‐way ANOVA followed by the Tukey's test. *P < 0.05, **P < 0.01 *vs*. control group, ^#^ *P* < 0.05, ^##^ *P* < 0.01 *vs*. soman group.

When 4‐PBA was applied, TUNEL staining showed a clear decrease in fluorescence intensity when compared to independent soman exposure (Fig. 4A). The mean fluorescence intensity of the 4‐PBA treatment group appeared to be lower than that of the independent soman exposure group (Fig. 4B). Western blot was also utilized to determine the effects of 4‐PBA. The findings demonstrated 4‐PBA significantly decreased the levels of GRP78 protein in comparison to the soman group, whereas the expression levels of ATF6 and CHOP proteins showed a downward trend, although this decrease was not statistically significant compared to the soman group (Fig. 4C–H).

### Soman induced ER stress in rats

**Fig. 5:**
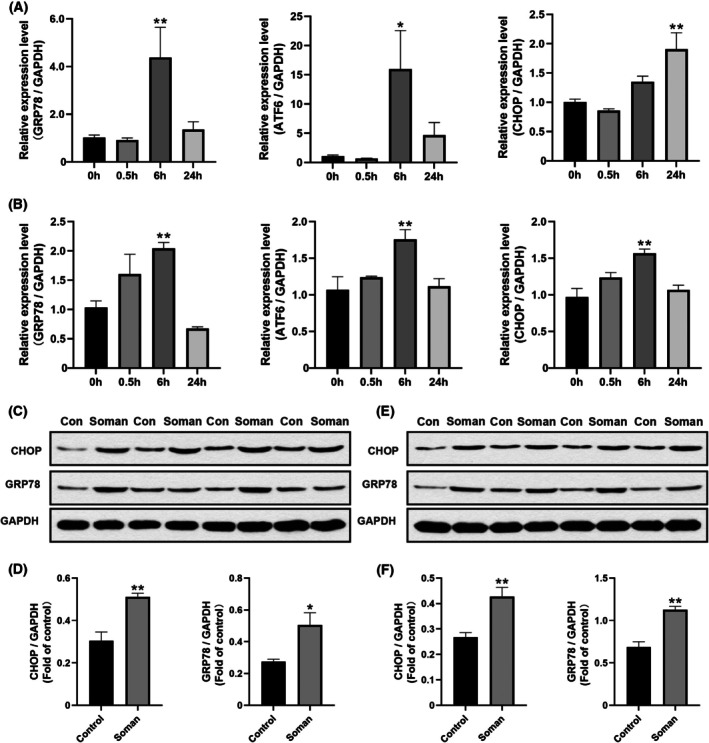
ER stress induced by soman in rats' brains. Changes of ER stress related mRNAs levels in hippocampus (A) and striatum (B) after soman exposure. Data are represented as the mean ± SEM and *n* = 6 for each group. Statistical analysis utilized one‐way ANOVA followed by the Tukey's test. *P < 0.05, **P < 0.01 *vs*. control group. Changes of CHOP and GRP78 protein in hippocampus (C) and striatum (E) of rats followed by soman exposure. The densitometric analysis of CHOP and GRP78 in hippocampus (D) and striatum (F). Data are represented as the mean ± SEM and *n* = 4 for each group. Statistical analysis utilized unpaired *t*‐test. *P < 0.05, **P < 0.01 *vs*. control group.

Our studies have shown that soman induces ER stress in cerebral organoids. Then we confirmed ER stress induction by soman in rats. As shown in Fig. 5A, the mRNA levels of key ER stress markers (GRP78, ATF6 and CHOP) in the hippocampus of rats significantly increased 6 h after soman exposure. Specifically, ATF6 and GRP78 levels increased significantly at 6 h, whereas CHOP levels increased by 89.21% at 24 h after exposure. In the striatum, ATF6, GRP78 and CHOP levels increased significantly by 101.02%, 97.67% and 61.42%, respectively (Fig. 5B). Notably, most mRNA levels at 24 h were lower than at 6 h, possibly as a result of the steady‐state regulation mechanism of ER stress [25].

To further verify ER stress induction by soman *in vivo*, we measured GRP78 and CHOP protein levels in the hippocampus (Fig. 5C,D) and striatum (Fig. 5E,F) of rats. The results showed significant increases in GRP78 and CHOP in the soman‐treated groups compared to the control group, which were consistent with the result in cerebral organoids.

## Discussion

Recent studies have highlighted the role of ER stress in the cytotoxicity induced by OPs. For instance, the pesticide malathion upregulated ER stress markers, including ATF6, PERK, IRE1, GRP78 and CHOP, in rat liver and kidney tissues [11]. In SH‐SY5Y neuroblastoma cells, tris(1,3‐dichloro‐2‐propyl) phosphate induced apoptosis accompanied by ER stress activation. Treatment with phenyl butyric acid reduced tris(1,3‐dichloro‐2‐propyl) phosphate‐induced ER stress and protected SH‐SY5Y cells from apoptosis by inhibiting Bax expression and increasing Bcl‐2 expression [26]. Bis(isopropyl methyl)phosphonate, which is structurally and toxicologically similar to sarin, caused cell death in human astrocytoma CCF‐STTG1 cells through ER stress activation [27]. Chlorpyrifos was shown to enhance the expression of ER stress‐related genes and proteins in mouse primary cortical neuronal cultures [13]. However, most of these findings were observed *in vitro* or in neuronal tissues applied *in vitro*; there is limited evidence of OP‐induced ER stress activation in brain tissues *in vivo*. Our study provided direct evidence that soman induced ER stress in the hippocampus of rats, as indicated by the significant increases in ATF6, GRP78 and CHOP expression 6 h after soman exposure.

Cells and animals are commonly used models for studying toxicity. However, 2D cultured cells fail to replicate the complex physiological structure and function of the human brain. Differences in anatomy, physiology and biochemistry between animals and humans may also lead to inaccurate results. Cerebral organoids generated from iPSCs resemble the human brain in terms of development, tissue structure and physiological function, making them advanced models for simulating the human brain in various studies.

Cerebral organoids have been increasingly used to evaluate the neurotoxicity of agents such as alcohol [28], graphene oxide [29], di‐(2‐ethylhexyl)phthalate [30], arsenic, lead, cadmium [31] and tetrodotoxin [32]. Organoids derived from peripheral blood mononuclear cells of veterans exposed to OPs in the 1991 Gulf War reflected the increased neurotoxic effects and demonstrated their potential as tools for personalized treatment [33]. ER stress has also been explored in cerebral organoids, for example, graphene oxide activated the IRE1α protein, leading to upregulation of XBP‐1. The ER stress inhibitor 4‐PBA prevented the GRP78‐IRE1α‐XBP‐1 axis activation induced by graphene oxide [29].

Given the advancements and applicability, we explored soman‐induced neurotoxicity in cerebral organoids. First, we examined the effects of soman on AChE activity in cerebral organoids because AChE is the primary target of OPs responsible for symptoms and fatalities. Soman inhibited AChE activity in a concentration‐dependent manner, consistent with previous *in vitro* and *in vitro* studies. Subsequently, TUNEL assays confirmed a significant increase in cellular apoptosis within cerebral organoids following soman treatment, emphasizing the direct neurotoxic effects of soman. Next, we tested ER stress biomarkers to confirm soman‐induced ER stress activation in cerebral organoids. Immunofluorescence results showed significant increases in the expression of GRP78 and CHOP after 200 nmol·L^−1^ soman exposure for 6 h. Quantitative PCR and western blot analyses further demonstrated significant increases in the mRNA and protein levels of GRP78, ATF6 and CHOP, indicating ER stress activation. Therefore, we pretreated cerebral organoids in soman exposure using 4‐PBA, the ER stress inhibitor. The TUNEL staining clearly showed a reduction in cell apoptosis. Next, we utilized western blot to assess the expression level of important proteins related to ER stress. Notably, GRP78, ATF6 and CHOP expression decreased in comparison to groups that did not receive 4‐PBA treatment, indicating that 4‐PBA suppressed ER stress and ultimately reduced cell apoptosis. These findings provide more evidence that soman triggered the ER stress pathway, which in turn caused cell apoptosis and confirmed in rat models.

The endoplasmic reticulum performs multiple functions, including lipid synthesis, Ca^2+^ homeostasis, and protein folding, processing and synthesis, all of which are crucial for maintaining cell homeostasis. It provides a highly specific environment that permits appropriate protein folding, making it vulnerable to toxic insults and stressful situations [34]. Accumulation of unfolded proteins in the ER, known as ER stress, occurs when cells are subjected to conditions such as decreased Ca^2+^ homeostasis, suppression of protein modification or degradation, and redox changes in the ER [35, 36]. Previous evidence has shown that soman poisoning rapidly inhibits cerebral AChE, resulting in a buildup of ACh in the brain. This elevation activates muscarinic receptors and causes a massive release of glutamate [37, 38]. Released glutamate activates N‐methyl‐D‐aspartic acid receptors, leading to excess free radical molecules or a buildup of intracellular Ca^2+^, initiating ER stress and subsequent neuropathology. The ER stress response, also called the unfolded protein response (UPR), is a cell adaptive mechanism involving three ER sensors: ATF6, PERK and IRE1. Under steady‐state conditions, GRP78 binds to these sensors, keeping them inactive. When stimulated by OPs, GRP78 dissociates from the sensors because of its higher affinity for misfolded or unfolded proteins, disrupting ER balance [39]. Long‐term UPR activation can lead to apoptotic cell death by upregulating CHOP, influenced by upstream PERK, ATF6 and IRE1. GRP78, a crucial ER stress marker, showed significant changes in our study, indicating ER stress occurrence in cerebral organoids.

**Fig. 6:**
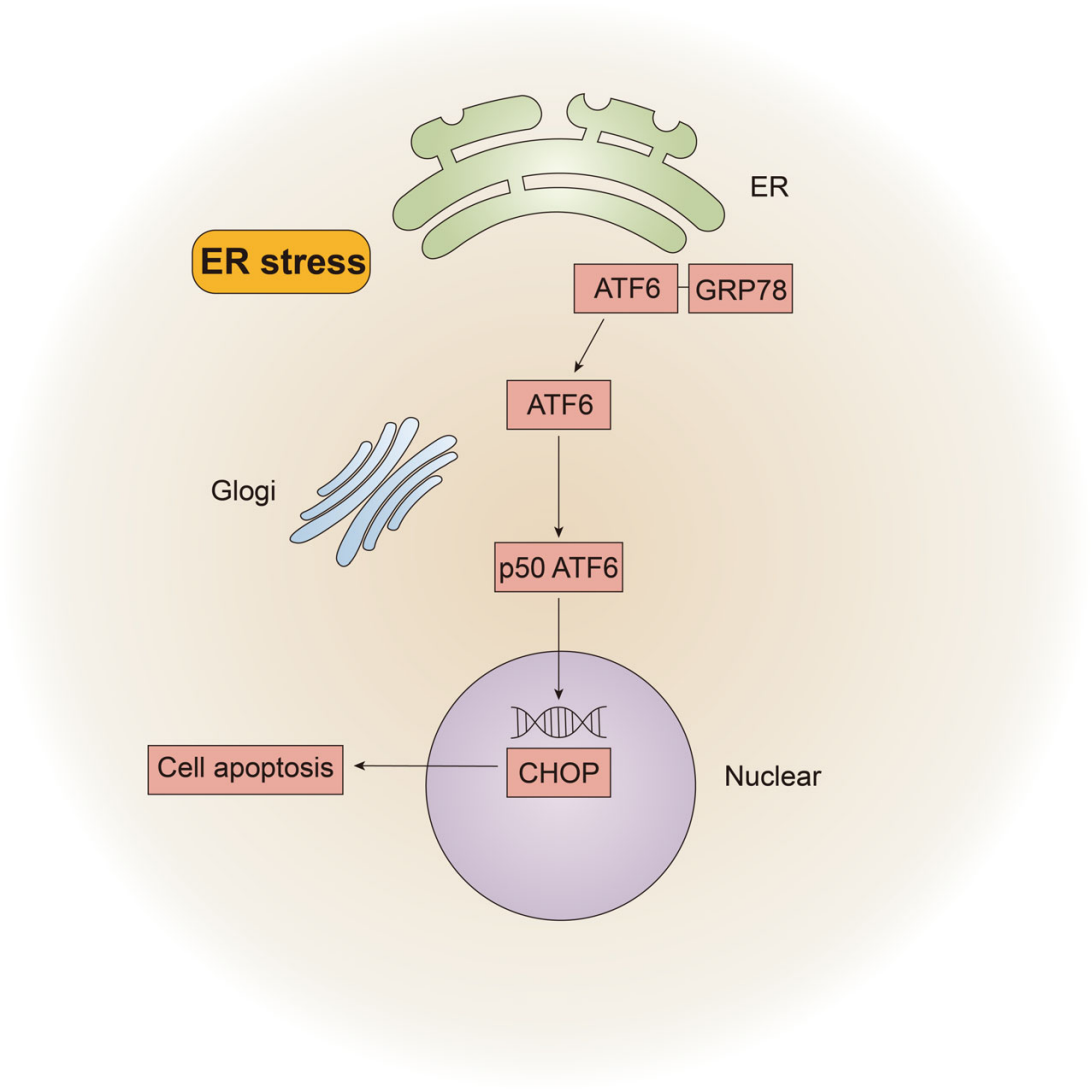
Schematic model for ATF6/GRP78/CHOP pathway in ER stress. ATF6‐GRP78 were bound on ER. The polymers split upon soman activation, and ATF6 was carried into the Golgi apparatus where it was cleaved. The ATF6 fragment entered the nucleus, where it increased CHOP gene expression and ultimately caused cell death.

Our results demonstrated that soman exposure leads to GRP78 binding to unfolded and misfolded proteins, releasing ER sensors, including ATF6, and activating the UPR (Fig. 6). ATF6 is cleaved in the Golgi apparatus and transported to the nucleus, promoting the expression of target genes, including CHOP [25]. Although GRP78 upregulation attempts to restore balance, excessive unfolded proteins result in persistent UPR activation, leading to CHOP upregulation and apoptosis [40, 41]. Notably, although GRP78 and ATF6 expression levels consistently increased at three time points, CHOP levels did not always align, possibly as a result of ER stress balance recovery. Continuous soman exposure for 24 h prevented GRP78 from restoring protein balance, leading to sustained UPR activation, CHOP upregulation and apoptosis.

The present study provides important molecular insights into how soman affects nervous system function by activating the ER stress pathway, offering a target for precaution and treatment for the neurotoxicity induced by soman. Currently, there are relatively few studies on OP‐induced neurotoxicity using 3D neuronal organoid models. Furthermore, this present showed a good use for iPSC‐derived cerebral organoids because it provided evidence indicating that soman induces correlated toxic effects through ER stress. With further research, 3D cerebral organoid models are expected to become crucial tools for evaluating OP neurotoxicity. We have observed cerebral organoids differentiated into specific brain regions, suggesting the potential for verifying the reliability of this tool across different brain regions. However, because of the absence of a blood–brain barrier, blood vessels and other essential tissues, our study results may differ from animal and clinical findings, necessitating the development of more complex organoid models.

## Conflicts of interest

The authors declare that they have no conflicts of interest.

## Author contributions

YW, XC and LL contributed to conceptualization. ZL contributed to data curation. QJ and WC contributed to investigations. YW and JS contributed to methodology. LL contributed to supervision. XC contributed to validation. YW, XC and LL contributed to writing the original draft. HC contributed to reviewing and editing.

## Supporting information


**Table S1.** Antibodies information.
**Table S2.** Primer sequences for qPCR.

## Data Availability

The data that support the findings of this study are available from the corresponding author upon reasonable request.
