# Supplementary material for: Soman induces endoplasmic reticulum stress and apoptosis of cerebral organoids via the GRP78‐ATF6‐CHOP signaling pathway
Source: FEBS Open Bio. 2025 Mar 28;15(7):1041–53. doi: 10.1002/2211-5463.70027 (PMC12226415; doi:10.1002/2211-5463.70027)
Supplement: Supplementary file 1 — Table S1. Antibodies information. Table S2. Primer sequences for qPCR. [file FEB4-15-1041-s001.docx]

**Soman induces endoplasmic reticulum stress and apoptosis of cerebral organoids via the GRP78-ATF6-CHOP signaling pathway**

Yue Wei^1,2^, Zhanbiao Liu^2^, Jingjing Shi^2^, Qian Jin^2^, Wenqian Chen^2^, Xuejun Chen^2*^, Liqin Li^2*^, Hui Chen^1,3*^

^1*^College of Pharmacy, Guilin Medical University, Guilin 541199, China

^2*^State Key Laboratory of NBC Protection for Civilian, Beijing 102205, China

^3*^Key Laboratory of Pharmacology for Prevention and Treatment of High Incidence Diseases in Guangxi Higher Education Institutions, Guilin Medical University, Guilin 541199, China

**Supplement Material**

**Supplement Table 1.** Antibodies information

| Antibody | Company | CAT | Dilute Ratio |
| --- | --- | --- | --- |
| Anti-SOX2 antibody | Abcam | ab92494 | 1:200 |
| Anti-Oct4 antibody | Abcam | ab181557 | 1:200 |
| Dylight 488 Goat Anti-Rabbit IgG | Abbkine | A23220 | 1:200 |
| Alexa Fluor 594 Goat Anti-Mouse IgG | Bioss | Bs-0296G-AF594 | 1:200 |
| Anti-GALC antibody | Abcam | ab232972 | 1:200 |
| Anti-GFAP antibody | Abcam | ab10062 | 1:200 |
| Recombinant Anti-MAP2 antibody | Abcam | ab254143 | 1;300 |
| Anti-beta III Tubulin antibody | Abcam | ab18207 | 1:200 |
| Anti-FOXG1 antibody | Abcam | ab18259 | 1:200 |
| Recombinant Anti-Reelin antibody | Abcam | AB312310 | 1:200 |
| TBR1 Polyclonal Antibody | Invitrogen | PA5-117379 | 1:300 |
| PAX6 Monoclonal Antibody | Invitrogen | MA1-109 | 1:200 |
| CTIP Monoclonal Antibody | Invitrogen | MAP5-31646 | 1:200 |
| SATB2 Monoclonal Antibody | Invitrogen | MA5-32788 | 1:100 |

**Supplement Table 2.** Primer sequences for qPCR

| **Gene** | **Forward Primer Sequence (5’-3’)** | **Reverse Primer Sequence (5’-3’)** |  |
| --- | --- | --- | --- |
| *ATF6* | AACAAGACCACAAGACCAA | AGGAGGAACTGACGAACT |  |
| *GRP78* | TGTTCAACCAATTATCAGCAAACTC | TTCTGCTGTATCCTCTTCACCAGT |  |
| *CHOP* | CTCTGGCTTGGCTGACT | TCTTCCTCCTCTTCCTCCT |  |
| *GAPDH* | CCGGGAAACTGTGGCGTGATG | AGGTGGAGGAGTGGGTGTCGCTGTT |  |
